# Effectiveness of an Inpatient Movement Disorders Program for Patients with Atypical Parkinsonism

**DOI:** 10.1155/2012/871974

**Published:** 2011-11-10

**Authors:** Anna D. Hohler, Jyeming M. Tsao, Douglas I. Katz, T. Joy DiPiero, Christina L. Hehl, Alissa Leonard, Valerie Allen, Maura Gardner, Heidi Phenix, Marie Saint-Hilaire, Terry Ellis

**Affiliations:** ^1^Department of Neurology, Boston University School of Medicine, 720 Albany Street, Suite 7B, Boston, MA 02118, USA; ^2^Braintree Rehabilitation Hospital, Braintree, MA 250 Pond Street, Braintree, MA 02184, USA; ^3^Department of Physical Therapy, College of Health & Rehabilitation Sciences, Boston University, 635 Commonwealth Avenue, Boston, MA 02215, USA

## Abstract

This paper investigated the effectiveness of an inpatient movement disorders program for patients with atypical parkinsonism, who typically respond poorly to pharmacologic intervention and are challenging to rehabilitate as outpatients. Ninety-one patients with atypical parkinsonism participated in an inpatient movement disorders program. Patients received physical, occupational, and speech therapy for 3 hours/day, 5 to 7 days/week, and pharmacologic adjustments based on daily observation and data. Differences between admission and discharge scores were analyzed for the functional independence measure (FIM), timed up and go test (TUG), two-minute walk test (TMW), Berg balance scale (BBS) and finger tapping test (FT), and all showed significant improvement on discharge (*P* > .001). Clinically significant improvements in total FIM score were evident in 74% of the patients. Results were similar for ten patients whose medications were not adjusted. Patients with atypical parkinsonism benefit from an inpatient interdisciplinary movement disorders program to improve functional status.

## 1. Introduction

Atypical parkinsonism is used to describe disorders characterized by parkinsonism—tremor, rigidity, akinesia, and postural instability—but not caused by Parkinson's disease (PD). These disorders often include other prominent features. The term includes progressive supranuclear palsy (PSP), multiple system atrophy (MSA), Lewy body dementia (LBD), corticobasal degeneration (CBD), vascular parkinsonism, drug-induced parkinsonism, and parkinsonism secondary to infection and other causes. PSP is characterized by parkinsonism along with a supranuclear vertical gaze palsy and early onset of balance problems and falls. The hallmark features of MSA include parkinsonism, autonomic instability, and cerebellar and corticospinal deficits. LBD has similar pathology to PD; however, accumulation of Lewy bodies in areas outside the substantia nigra leads to hallucinations, cognitive impairment and dementia prior to the onset of parkinsonism. Features of CBD include asymmetric parkinsonism, apraxia, alien limb phenomenon, aphasia, and sensory deficits. Vascular parkinsonism is due to lacunar infarcts in the basal ganglia and can be distinguished from PD by an abrupt onset or stepwise deterioration and development of parkinsonism and evidence of neurovascular disease. Drug-induced parkinsonism is often due to antipsychotics or antiemetics and usually resolves with cessation of the offending drug [1, 2].

Treatment of PD involves medications that increase dopamine in the basal ganglia. However, there has been less success with pharmacologic treatment in atypical parkinsonism [1, 3–5]. Previous studies have shown physical therapy to be an effective adjunctive treatment in patients with PD [6, 7] but there have only been a handful of case reports and studies investigating the efficacy of nonpharmacologic therapy in atypical parkinsonism patients.

Case reports and studies have shown subjective and objective improvements in gait, balance, and patient safety in patients with PSP [8–12] and in a patient with mixed CBD and PSP [13]. Similar improvements in gait, balance, transfers, and stability were seen in case reports of physical therapy intervention in patients with MSA [14, 15]. Timed up and go, functional reach test, 360-degree turn, and 50 foot timed walk are examples of some of the improved objective measures. Another case report showed improvement in activities of daily life (ADLs) and finger manipulation after repetitive finger exercises in a patient with CBD [16]. A small pilot randomized controlled trial showed significant improvement in MSA patients after receiving individualized outpatient occupational therapy [17].

Regarding intensive inpatient programs, a prior study by Ellis et al. investigated the effectiveness of an inpatient rehabilitation program for people with PD. In the study, medication was adjusted and interdisciplinary rehabilitation program was provided to optimize patients' functional ability. Significant improvements were noted in all outcome measures. Patients who did not have changes made to their medications also showed significant improvements in total, motor, and cognitive functional independence measure (FIM) scores [18].

The purpose of this study was to investigate the effectiveness of an inpatient movement disorders program in improving functional status for patients with atypical parkinsonism and to determine whether or not these findings were clinically meaningful. We hypothesized that people with atypical parkinsonism would show statistically and clinically significant improvements in functional status after participating in such a program.

## 2. Methods

### 2.1. Design and Subjects

A pretest-posttest design was used to determine the effectiveness of a movement disorder program for patients admitted to an inpatient rehabilitation hospital with the diagnosis of atypical parkinsonism. Patients were admitted from home, acute care facilities, skilled nursing facilities, or assisted living between January 2004 and August 2008. They carried diagnoses, determined by a neurologist specializing in movement disorders, which fall under the term “atypical parkinsonism” as described above. They were at least 18 years old and were given a Hoehn and Yahr stage I to V for classification of PD. A total of 91 subjects were admitted to the program. Baseline characteristics are listed in Table 1.

### 2.2. Outcome Measures

Primary outcomes were FIM total score. Secondary outcomes included FIM motor score, FIM cognitive score, 2-minute walk test (TMW), Timed “up and go” test (TUG) Berg balance score, and finger tapping test (FT).

The FIM is a widely used 18-item assessment of disability among inpatient rehabilitation patients. The FIM measures ability to perform basic life activities, such as self-care, sphincter control, transfers, locomotion, communication, and social cognition. Each item is scored on a scale from 1 to 7, in which 1 is patient requires total assistance to complete the task and 7 is complete independence. The FIM can be divided into 2 sections: motor (13 items) and cognitive (5 items). It has been shown to have good reliability and validity [19].

The TMW is performed by asking subjects to walk as far as they can in 2 minutes. Patients with PD have been shown to cover less distance than age-matched controls [20].

The TUG assesses a patient's ability to transfer from sitting to standing, ambulate, and make a turn. Patients are timed while rising from a chair, walking 3 m, turning, walking back to the chair, and sitting down. It has been shown to have high interrater reliability for subjects with PD [21]. Subjects were allowed to use an assistive device if necessary for the TMW and the TUG.

The BBS is a 14-item scale assessing balance while sitting, standing, turning, and reaching forward. Items are rated from 0 to 4, with 0 meaning the subject needs assistance or is unable to perform the task and 4 meaning the subject can perform the task safely and independently. It has been shown to be reliable and valid in patients with PD [22, 23]. Minimal detectable change (MCD) has been found to be +/−6 points among patients who have suffered a stroke [24] and +/−5 points in patients with PD [25].

The FT is a timed test useful in assessing the impact of bradykinesia on rapid alternating movements of the upper extremity. Two buttons are attached to a counter 30 cm apart. Subjects are asked to alternate tapping each button with their left hand for one minute. The sum of the taps is the score for that hand. The test is repeated with the right hand. The FT has been shown to have good validity and reliability and is able to distinguish normal subjects from those with PD [26].

### 2.3. Intervention

A multidisciplinary team consisting of neurologists specializing in movement disorders and neurorehabilitation, physical therapists, occupational therapists, speech-language pathologists, nurses, and case managers provided a comprehensive rehabilitation program for patients admitted to the hospital.

All outcome measures were obtained at admission and discharge, as well as daily measurements of TMW and TUG at the peak and troughs of medication cycles. The same therapists administered interventions and outcome measures. Weekly rounds were conducted to allow the whole team to evaluate the data and discuss the patients' status. Decisions regarding changes to medications or rehabilitation interventions were made at this time. Subjects' responses to medication adjustments were discussed further at weekly movement disorder meetings. Adjustments were made to subjects' therapy and medications during the entire length of stay at the hospital. Medication adjustments included increases or decreases in Parkinson's disease medications to optimize peak performance. Those medications included carbidopa/levodopa, monoamine oxidase inhibitors, catechol-o methyltransferase inhibitors and amantadine.

Subjects received individually tailored physical, occupational, and speech therapy for a minimum of 3 hours per day for 5 to 7 days per week. Therapy was provided on an individual and group basis. Interventions included external cueing to improve gait speed, step length and cadence [27–30], cognitive movement strategies during task-based training to improve mobility, balance and transfers [6, 31–33], resistive exercises [34], exercises for joint mobility [35, 36], and speech therapy to improve voice volume and clarity [37, 38]. A more detailed description of the intervention is provided in Table 2. 

### 2.4. Data Analysis

Means, standard deviations, and frequency distributions were calculated for subjects' baseline characteristics, length of stay, and disposition. The efficacy of the intervention was evaluated by comparing admission and discharge mean scores for each of the outcome measures. Two-tailed paired *t* tests were conducted with an alpha level set at 0.05. A Bonferroni-adjusted type I error rate (*α* = 0.007) was applied to all *t* tests. Results were calculated for patients who received rehabilitation along with PD medication adjustments and for patients who received rehabilitation only. Clinically significant improvement was determined based on a total FIM score change of ≥22, which has been associated with the minimal clinically important difference in people who have suffered a stroke [39]. In this study a change from admission of more than 22 was considered to be clinically meaningful.

## 3. Results

### 3.1. Subjects

Ninety-one subjects with atypical parkinsonism underwent rehabilitation therapy. Average age at admission was 76.5 years (SD 0.81), and they had been carrying the diagnosis of parkinsonism for an average of 5.7 years (SD 0.55). Of the 91 subjects with atypical parkinsonism, 25 (27.5%) had vascular parkinsonism, 19 (20.9%) had MSA, 4 (4.4%) had PSP, and the remaining cases were either medication related, due to LBD, CBD, toxin exposure or were unknown. Eighty-five of the subjects were Hoehn and Yahr stages III-V. Six subjects were not evaluated using Hoehn and Yahr (Table 1). The rehabilitation team made changes to 81 subjects' medications while receiving physical therapy. Ten subjects underwent physical rehabilitation only with no changes to their medications. Length of stay varied from one to six weeks with an average stay of 2.5 weeks.

### 3.2. Outcomes for All Patients

Statistically significant improvements were made in all outcome measures over the course of the rehabilitation program (Table 3). Total FIM score increased 29.5 points (95% CI = 26.4–32.5). Additionally, motor FIM improved 25.9 points (95% CI = 23.4–28.5) and cognitive FIM improved 3.5 points (95% CI = 2.6–4.4). TUG decreased by 39.4 seconds (95% CI = 20.6–58.2), TMW lengthened by 63.5 feet (95% CI = 44.3–82.9), and Berg balance scale scores improved 7.5 points (95% CI = 4.3–10.6). Left and right finger tapping improved by 11.5 taps (95% CI = 6.7–16.1) and 10.8 taps (95% CI = 5.8–16.1), respectively.

Previous studies have shown the minimal clinically important difference of total FIM to be 22 [39]. Using this cutoff, sixty-five (74%) patients made clinically meaningful improvements in total FIM.

### 3.3. Outcomes for Patients Receiving Rehabilitation Only and No Changes to Medication

Statistically significant improvements were made in all but left finger tapping for the 10 patients who received rehabilitation only (Table 4). Analysis showed an improvement of 32.1 (95% CI = 22.8–41.3) for total FIM, 28.6 (95% CI = 19.8–37.3) for motor FIM, and 3.5 (95% CI = 1.7–5.2) for cognitive FIM. TUG decreased by 52 seconds on average (95% CI = 13.1–91.0), TMW length increased by 76 feet (95% CI = 27.4–124.5), and right finger tapping improved by 19.6 taps (95% CI = 3.4–25.7). Left finger tapping increased on average 7.8 taps, but results were not statistically significant (95% CI = −9.7–25.4).

## 4. Discussion

This study investigated the effectiveness of an interdisciplinary inpatient rehabilitation program for patients with atypical parkinsonism. Our results showed improvements in total FIM, motor FIM, cognitive FIM, TMW, TUG, BBS, and left and right FT. Among patients who received rehabilitation only, without changes to their medication regimens, statistically significant improvement in all but left FT was observed. Clinically meaningful improvement, defined by a change in total FIM of greater than 22, was also observed in 74% of patients. These results imply that patients with atypical parkinsonism can show significant improvement in function after receiving intensive inpatient multidisciplinary therapy including rehabilitative and pharmacologic interventions.

Patients with atypical parkinsonism are difficult to manage on an outpatient basis. The complexity of their symptoms, the added cognitive and autonomic deficits, the poor response to most PD pharmacological agents, and the relatively rapid decline in status contribute to the challenges in managing these patients particularly as the disease progresses. This study highlights the benefits of an interdisciplinary rehabilitation program in addition to medication adjustments in an inpatient setting, where patients could be observed over a 24-hour period, 7 days per week by health care professionals with expertise in movement disorders. Objective measures taken daily during peak and troughs of medication cycle allowed an objective, systematic assessment of function. This data was used to guide the decision-making process regarding pharmacological adjustments and the focus of rehabilitation strategies.

Strengths of our study include the large sample size, the variety of disorders, and the advanced disability stages of the patients. Other studies investigating the effectiveness of rehabilitation have been case reports, small pretest-posttest trials with a sample size of 19 or less, and a pilot RCT with a sample size of 17 [6, 8–10, 12–15]. In addition, most of the studies included subjects with PSP, whereas our study sampled across categories of atypical parkinsonism and included primarily vascular parkinsonism and MSA. Our patients were also at higher levels of disability and all were Hoehn and Yahr stages III to V, suggesting that functional gains can be made in these patients with complex symptoms in later stages of the disease.

Limitations of the study include lack of a control group and prescribing rehabilitation while simultaneously changing medications. We are unable to distinguish what effects were due purely to physical rehabilitation. However, our smaller group of 10 patients who received rehabilitation only did show significant improvement, but larger studies should be conducted to address the particular effects of rehabilitation alone. It is also difficult to assess whether the intensive nature of the program itself had any effect on the subjects' improvement. The higher frequency of assessments and therapy sessions in an inpatient setting may have continued to the improvements observed. Factors such as treatment intensity, the availability of objective data for treatment decisions and goal setting, and the expertise and frequent communication of the interdisciplinary team were not individually assessed. Further studies comparing specialized interdisciplinary movement disorder rehabilitation programs such as the program described in this study with more traditional standard rehabilitative care would help clarify this question.

Another limitation of our study is the lack of long-term followup data. It is unknown if the gains made during the rehabilitation admission were sustained following discharge. One study of patients with idiopathic PD in Hoehn and Yahr stages II and III who participated in an inpatient exercise training and muscle strengthening program sustained improvements in quality of life at follow-up [40]. Lastly, the gains in the total FIM used to assess clinically meaningful improvement were extrapolated from the stroke literature [39], as this has not been derived in parkinsonian patients.

While our study demonstrated that patients with atypical parkinsonism can benefit from an intensive inpatient rehabilitation program, further studies are needed to look at the long-term gains. In addition, research is needed to assess the efficacy of inpatient rehabilitation programs on atypical parkinsonism patients with earlier stages of disease and their effect on the progression of their disorders.

## Figures and Tables

**Table 1:** Patient baseline characteristics.

Characteristic	
Age—y, mean (SD) *n* = 91	76.6 (7.7)
Disease duration—y, mean (SD) *n* = 91	5.7 (4.4)
Sex no. (%) men/women *n* = 91	53 (58.2)/38 (41.8)
Race no. (%) white *n* = 91	84 (92.3)
Education no. (%) ≤ bachelor's/>bachelor's *n* = 75	51 (56)/24 (26.4)
Hoehn and Yahr stage no. (%) *n* = 85	
I	0 (0)
II	0 (0)
III	26 (28.6)
IV	43 (47.3)
V	16 (17.6)
Length of stay—d, mean (SD)	24.4 (11.9)

Diagnosis no. (%) *n* = 91^a^	
Vascular parkinsonism	25 (27.5)
MSA	19 (20.9)
PSP	4 (4.4)
Medication related	2 (2.2)
LBD	1 (1.1)
CBD	1 (1.1)
Toxin	1 (1.1)
Unknown	38 (41.8)

^a^MSA: multiple system atrophy, PSP: progressive supranuclear palsy, LBD: Lewy body dementia, and CBD: corticobasal dementia.

**Table 2:** Description of interventions.

Functional training	Rolling from supine position to sitting position and from sitting position to supine position.
	Transferring from sitting position to standing position, from chair to bed, and from chair to toilet.
	Dressing and grooming.
	Balance: reactive and anticipatory within functional contexts.

Gait training	Walking with external auditory cues from a metronome to optimize gait speed and cadence; increasing cadence by 10% over baseline and progressing until cadence approaches normal or until subject reaches maximum capacity.
	Reducing freezing (context specific: doorways, thresholds, and narrow spaces) with visual cues in the form of lines on the floor from tape or laser beams.
	Reducing freezing (context specific: doorways, thresholds, and narrow spaces) with visual cues in the form of lines on the floor from tape or laser beams.
	Improving adaptation (various walking surfaces, obstacles in the environment, starting and stopping, and turning head while walking).
	Curb negotiation and stair climbing.

Range of motion, flexibility, strengthening exercises	Range of motion (increase trunk extension and rotation).
	Stretching (hip flexor, hamstring, and gastrocnemius muscles).
	Strengthening (trunk and hip postural muscles and knee and ankle extensor muscles).

Speech exercises	Exercises to improve vocal rate control.
	Exercises to improve phonation.

**Table 3:** All patients.

Measure (*n*)^a^	Admission mean (SD)	Discharge mean (SD)	Change (95% CI)
Total FIM (88)	41.3 (15.7)	70.8 (21.4)	29.5 (28.4, 32.5)
Motor FIM (88)	23.6 (11)	49.6 (16.6)	25.9 (23.4, 28.5)
Cognitive FIM (87)	17.7 (6.1)	21.2 (5.8)	3.5 (2.6, 4.4)
TUG (60)	81.5 s (89.4)	42.0 s (46.9)	−39.4 s (−20.6, −28.2)
TMW (60)	138.9 ft (76.9)	202.5 ft (96.9)	63.5 ft (44.2, 82.9)
BBS (28)	22 (12.2)	29.5 (13.4)	7.5 (4.3, 10.6)
Left FT (51)	60.2 (23.3)	71.7 (24.8)	11.5 (6.7, 16.1)
Right FT (50)	68.3 (27)	79.3 (30)	10.9 (5.8, 16.1)

^a^FIM: functional independence measure, TUG: timed up and go, TMW: 2-minute walk, BBS: berg balance scale, and FT: finger tap.

**Table 4:** Patients receiving rehabilitation only.

Measure (*n*)^a^	Admission mean (SD)	Discharge mean (SD)	Change (95% CI)
Total FIM (10)	38.3 (13.4)	70.4 (23.4)	32.1 (22.8, 41.3)
Motor FIM (10)	21.2 (7.8)	49.6 (17.6)	28.6 (19.8, 37.2)
Cognitive FIM (10)	17.1 (6.1)	20.6 (6.1)	3.5 (1.7, 5.2)
TUG (10)	83.4 s (61.5)	31.3 s (10.1)	−52.1 s (−13.7, −91.0)
TMW (9)	114.4 ft (77.6)	190.4 ft (68.4)	76.0 ft (27.4, 124.5)
Left FT (8)	61.5 (19.8)	69.3 (13.4)	7.8 (−9.7, 25.4)
Right FT (8)	67.2 (24.8)	86.8 (24.9)	19.6 (3.4, 35.7)

^a^FIM: functional independence measure, TUG: timed up and go, TMW: 2-minute walk, BBS: berg balance scale, and FT: finger tap.
